# Diabetes in Socioeconomically Vulnerable Populations

**DOI:** 10.1155/2015/247636

**Published:** 2015-12-21

**Authors:** Anil Kapur, Maria I. Schmidt, Alberto Barceló

**Affiliations:** ^1^World Diabetes Foundation, 2820 Gentofte, Denmark; ^2^Universidade Federal do Rio Grande do Sul, 90470-280 Porto Alegre, RS, Brazil; ^3^World Health Organization, Washington, DC 20037, USA

Diabetes mellitus (DM) is a global epidemic that is increasing rapidly. An estimated 415 million people worldwide currently live with DM and another 318 million people have impaired glucose tolerance (IGT), a marker for future DM. By 2040 these numbers are likely to grow to 642 million and 481 million, respectively. Over 75% of prevalent cases live in low and middle income countries. Economic transition (from extreme poverty to sustenance living), urbanization, technology, and globalization are changing the way we live and work. Societies in rapid transition show these changes most visibly; here lifestyles, diets, eating habits, and culture are changing rapidly with the changing urban landscapes and new economic realities. Traditional diets are being replaced by poor quality, relatively less expensive, easily accessible, highly processed food with more fat, salt, and sugar. This is paralleled by increased use of motorized transport and decrease in physical activity.

The mismatch between the predicted environment for survival programming and the actual environment in adult life may be a critical factor driving type 2 diabetes and obesity epidemic. Mounting evidence shows that prenatal and early life development influenced by parent's health, particularly the mother's body composition and nutritional and metabolic status during pregnancy, affect risk for noncommunicable diseases (NCDs) including diabetes in later life through fetal programming. This is especially relevant to low-resource countries. Studies on survivors of the Dutch and Chinese famine show that individuals exposed to intrauterine under nutrition had significantly higher rates of diabetes, with the risk being highest in the subgroup that were relatively well off in adult life. As millions are lifted out of abject poverty in China and India, diabetes rates have started rising dramatically with a lag of three to four decades in these countries.

Young women, born small as a consequence of their mothers' undernutrition during pregnancy, may have difficulty in coping with the insulin resistance and metabolic demands of pregnancy, resulting in hyperglycemia and higher rates of gestational diabetes mellitus (GDM). Hyperglycemia in pregnancy is associated with serious complications for both the mother and child contributing to poor pregnancy outcomes and maternal and neonatal morbidity and mortality. Maternal health and diabetes are closely linked; poor maternal nutrition and health increases vulnerability to hyperglycemia and hyperglycemia increases risk of poor pregnancy outcome as well as future risk of obesity and diabetes in both the mother and her offspring. GDM creates a vicious cycle in which diabetes begets diabetes. The cycle of vulnerability is repeated with increasing risk accumulation in subsequent generations.

Poor access to care particularly among the less fortunate and vulnerable sections of the society exaggerates the health and economic problem when diabetes appears. Studies show that uneducated, unemployed people, especially those living in semiurban or rural areas who cannot afford or do not have access to even bare minimum health care, are likely to be diagnosed late and likely to develop or have at presentation diabetes related complications. Financial status has been shown to be a strong predictor of diagnosis and effective management of diabetes. This has remarkable socioeconomic significance: those who need more advanced/more expensive care for diabetes related complications are the ones who can ill afford such care forcing many of them to borrow and enter the debt trap with disastrous consequences to the individual and society. Data suggests that poverty is a predictor of higher mortality among people with diabetes and the risk of dying among the poor is not completely explained by a higher frequency of chronic complications.

The rise of diabetes in the low and low-middle income countries has the potential of exaggerating another major public health problem affecting the poor and vulnerable sections of populations, the burden of tuberculosis. Diabetes is associated with a threefold increased risk of TB and increased risk of death during TB treatment.

Thus the overall impact of diabetes among the disadvantaged not only is a consequence of early life programing, social determinants, and higher exposure to risk factors but also is due to lower access to diagnosis and care for diabetes.

Although type 2 diabetes has been shown to be preventable, the long term impact of preventive strategies in the real world is still to be seen. The WHO Country Capacity Survey has reported that national diabetes policy plans and strategies for prevention and control programs are popular among low and middle income countries and even diabetes guidelines are available in most countries; but major gaps exist in the implementation as two-third of the countries report that guidelines are not operational or have no allocated resources for implementation.

The Sustainable Development Goals (SDGs) being launched in January 2016 will guide the development agenda, including health, up to 2030. Public health action to address the prevention and care for diabetes and other NCDs is included in SDG 3 (good health and well-being). Because of its link to maternal health and comorbidity with TB and other conditions, actions to address diabetes will require collaboration between maternal, newborn, and child health, noncommunicable diseases and communicable diseases, and seamless connection between health promotion, disease prevention, and care delivery, thereby strengthening overall health systems. These actions will also contribute to and benefit from action on several other SDG goals such as SDG 1 (ending poverty in all its forms), SDG 2 (ending hunger), SDG 5 (gender equality), SDG 6 (ensuring clean water and sanitation), SDG 10 (reducing inequalities), SDG 11 (making cities safe, resilient, and sustainable), and SDG 17 (strengthening global partnerships).

*Anil Kapur* *Anil Kapur* *Maria I. Schmidt* *Maria I. Schmidt* *Alberto Barceló* *Alberto Barceló*

